# Enhancing Postoperative Decision‐Making in Esophageal Cancer: Clinical Perspectives and Future Research Needs

**DOI:** 10.1002/cam4.71786

**Published:** 2026-05-10

**Authors:** Huazhen Wu, Jia Liao, Lishan Hu, Siwen Li

**Affiliations:** ^1^ Department of Tumor Chemotherapy The Qingyuan Affiliated Hospital of Guangzhou Medical University, Qingyuan People's Hospital Guangdong China; ^2^ Department of Radiotherapy Oncology The Qingyuan Affiliated Hospital of Guangzhou Medical University, Qingyuan People's Hospital Guangdong China; ^3^ Department of Health Management The Qingyuan Affiliated Hospital of Guangzhou Medical University, Qingyuan People's Hospital Guangdong China; ^4^ Department of Thoracic Surgery The Qingyuan Affiliated Hospital of Guangzhou Medical University, Qingyuan People's Hospital Guangdong China

To the Editor,

We read with great interest the recent article by Huang et al. [1], “Tumor Regression Grade as a Predictor of Adjuvant Therapy Benefits in Esophageal Squamous Cell Carcinoma Patients. After Neoadjuvant Therapy” published in *Cancer Medicine*. The authors should be commended for conducting a multicenter analysis with a relatively large cohort and long follow‐up. Their findings highlight the novel and clinically relevant role of tumor regression grade (TRG) as a stratification tool to guide the use of adjuvant therapy. In particular, the demonstration that patients with good pathological responses (TRG 0–1), as well as those with ypN+ and ypT3–4 disease, derive significant survival benefits is of high value for clinical decision‐making and may help refine current guideline recommendations.

Despite these important contributions, several limitations warrant further consideration. First, the study grouped different types of postoperative adjuvant therapy—chemotherapy and chemo‐immunotherapy—into a single category [2, 3]. Such heterogeneity may dilute or obscure true differences in efficacy. Future studies should stratify outcomes based on regimen type and treatment duration to clarify which therapeutic combinations provide the greatest benefit in specific TRG‐defined subgroups.

Second, the retrospective design raises concerns of immortal time bias and selection bias. Patients who were eligible and fit enough to receive adjuvant therapy are inherently different from those who were not, and the lack of methods such as landmark analysis or time‐dependent covariates may overestimate the survival advantage. A prospective design, or at minimum advanced causal inference methods such as target trial emulation, would help strengthen the validity of these findings.

Third, TRG evaluation itself carries an element of subjectivity, even under standardized criteria. The absence of centralized pathological review or assessment of inter‐observer variability may introduce misclassification. Incorporating digital pathology and artificial intelligence‐based quantification of residual tumor burden could provide more objective and reproducible measurements. Additionally, combining TRG with molecular biomarkers such as ctDNA or PD‐L1 expression may enhance predictive accuracy and support a more comprehensive framework for adjuvant therapy decisions [4, 5].

In summary, Huang et al. provide valuable evidence that TRG can serve as a practical and clinically meaningful biomarker to identify esophageal squamous cell carcinoma patients who are most likely to benefit from adjuvant therapy after neoadjuvant treatment. While the study advances the field, future work should address regimen‐specific efficacy, adopt more robust analytic strategies to minimize bias, and integrate objective or molecular markers with TRG to refine risk stratification. We believe that these steps will further optimize postoperative management and ultimately improve outcomes for this challenging disease.

## Author Contributions

All authors contributed to the study conception and design. Study design, H.W., J.L., S.L., Writing – original draft, H.W., J.L., L.H.; Writing – review and editing, H.W., J.L., L.H.; Supervision: S.L.

## Funding

The authors have nothing to report.

## Conflicts of Interest

The authors declare no conflicts of interest.

## Data Availability

The manuscript is a commentary on previously published articles and does not involve any data.
